# Modelling and Predictive Monitoring of Business Processes under Uncertainty with Reinforcement Learning

**DOI:** 10.3390/s23156931

**Published:** 2023-08-03

**Authors:** Alexandros Bousdekis, Athanasios Kerasiotis, Silvester Kotsias, Georgia Theodoropoulou, Georgios Miaoulis, Djamchid Ghazanfarpour

**Affiliations:** 1 Department of Informatics and Computer Engineering, University of West Attica, 12242 Egaleo, Greece; 2 Department of Informatics, University of Limoges, 87032 Limoges, France

**Keywords:** predictive business process monitoring, process mining, business process management, machine learning, data analytics

## Abstract

The analysis of business processes based on their observed behavior recorded in event logs can be performed with process mining. This method can discover, monitor, and improve processes in various application domains. However, the process models produced by typical process discovery methods are difficult for humans to understand due to their high complexity (the so-called “spaghetti-like” process models). Moreover, these methods cannot handle uncertainty or perform predictions because of their deterministic nature. Recently, researchers have been developing predictive approaches for running business cases of processes. This paper focuses on developing a predictive business process monitoring approach using reinforcement learning (RL), which has been successful in other contexts but not yet explored in this area. The proposed approach is evaluated in the banking sector through a use case.

## 1. Introduction

Business Process Management (BPM) enables organizations to merge their own requirements with those of their customers, the company’s goals, and the monitoring and execution of business processes [1]. There is a growing trend in which information systems automatically capture and make process data available in the form of event logs. Process mining can be used to analyze business processes based on their recorded behavior in event logs, providing a means to discover, monitor, and improve processes across different application domains [2]. Proper application of BPM leads to enhanced efficiency and productivity while reducing costs and errors. BPM also offers a way for businesses to ensure the secure execution of procedures, protocols, resources, and capital management, as well as evaluate and optimize their own processes with ease [3].

The demand for data scientists who can transform data into valuable insights is rapidly increasing. In the context of process mining, the challenge is to extract relevant information about the actual processes being executed from the vast amount of data available. Process mining aims to discover, monitor, and improve real processes by extracting knowledge from event logs readily available in today’s information systems [4]. Therefore, process mining can analyze these data to establish a relationship between the observed behavior of people, machines, and organizations and the modeled behavior. The standard process discovery techniques may generate complex process models (the so-called “spaghetti-like” process models) and are difficult for humans to comprehend [5]. The complexity of the process models may depend on the event log behavior, the implemented process discovery algorithm, etc. Most importantly, these models are deterministic, so they are not capable of managing uncertainties that are implicit in business processes or generating predictions about their future execution [6]. Recently, there has been a focus on developing approaches for predictive business process monitoring to forecast the future progression of ongoing cases of a business process [6]. Predictive business process monitoring targets different goals, such as predicting the next activity to be performed and estimating its execution time or anticipating all the activities to be executed until the end of the trace, namely the trace suffix, as well as the total execution time of the trace, that is, the trace time [7,8].

In recent years, there has been a surge in research studies that suggest utilizing machine learning algorithms to enable predictive capabilities. However, the application of reinforcement learning (RL) in the field of business processes has been overlooked [8]. RL is a type of machine learning technique that trains models to maximize a reward signal without labeling data or attempting to uncover any underlying patterns [9].

The current paper is an extension of our previous work [10]. In this paper, motivated by previous successful implementations of RL in various contexts, we develop a predictive business process monitoring approach with the use of RL. More specifically, the proposed approach takes as input an event log and provides as output predictions about the next activities, and specifically about activities that have been defined as goal states (e.g., activities corresponding to decisions), along with the most efficient path on a process model. To do this, it incorporates five subsequent steps: (i) event log extraction; (ii) process discovery for generating options in process models; (iii) process statistical analysis for selection of the process model; (iv) handling incomplete traces; and (v) creating the uncertain process model and providing predictions about the business process. The proposed approach is evaluated in the context of a use case from the banking sector and is compared to deep learning algorithms.

The rest of the paper is organized as follows: Section 2 presents the theoretical background and the related works on process mining and predictive business process monitoring. Section 3 describes our proposed approach for the modelling and predictive monitoring of business processes under uncertainty with RL. Section 4 discusses the results derived from the deployment of the proposed approach to the banking sector and performs a comparative analysis with deep learning algorithms. Section 5 concludes the paper and presents our plans for future work.

## 2. Background and Related Works

Process mining extracts valuable insights and information from the event logs generated by various types of information systems [11]. It involves using specialized software tools to analyze event logs and discover the hidden patterns, structures, and correlations that exist within them [4]. By analyzing these patterns and structures, process mining can help organizations identify bottlenecks, inefficiencies, and other areas where processes can be improved [2].

Despite their proven benefits, process discovery methods usually create complex process models (the so-called “spaghetti-like” models) that are not easily understood by humans [12]. Moreover, due to their deterministic nature, they are not capable of handling uncertainty or performing predictions [6]. To this end, predictive business process monitoring methods have emerged.

Several research works deal with predictive business process monitoring with the use of machine learning methods for a variety of applications [2]. Machine learning methods that have been used include Naïve Bayes classifier [13], Support Vector Regression (SVR) [14], logistic regression [15], random forest [16], K-nearest neighbor [17], Bayesian Networks [6,14], trace clustering [18], and Artificial Neural Networks (ANN) [19]. In addition, predictive business process monitoring can benefit from deep learning methods. Long Short-Term Memory (LSTM) and Convolutional Neural Networks (CNN) have attracted increased research attention [20,21,22,23,24]. They have been used for predicting the next event of a running case and its timestamp [20,24]; predicting sequences of the next events and their associated resource pools [20]; predicting the remaining time [25]; and modelling the time dependencies between events [26]. Despite the promising results of deep learning methods in predictive business process monitoring, their explainability has arisen as a challenge [27,28,29], and they require vast amounts of data [30]. At the same time, predictive business process monitoring has not sufficiently adopted other machine learning algorithms. For a more detailed review of machine learning in predictive business process monitoring, the reader may refer to [15,31,32].

In this context, reinforcement learning (RL) in predictive process monitoring has just started to emerge [8] as a promising type of ML capable of training models to directly maximize a reward signal, without assigning any label or necessarily trying to find some hidden structure in the data. In RL, a learning agent tries to achieve a predefined goal state while navigating through an environment that consists of states and actions. The agent learns by receiving rewards for selecting actions that lead to different states and moves closer to the goal state. Unlike conventional instruction-based methods, RL involves learning by trial and error [9]. However, for RL to be effective in complex, real-world scenarios, agents need to efficiently represent high-dimensional inputs and generalize their past experiences to new situations, something which is a challenging task [33].

Despite the large number of successful implementations of RL in various contexts (e.g., [34,35,36,37]), its applicability in business processes is an underexplored area [8]. In [38], the authors implemented an RL-based approach for optimizing resource allocation in business process execution in order to tackle the complexity and dynamicity of resource allocation in BPM. The experimental results indicate that their approach outperforms well-known heuristic or hand-coded strategies. In [39], the authors proposed deep RL for business process optimization. More specifically, the author implemented deep Q-learning in order to deal with uncertain environments where business processes are executed. Their results showed that business process optimization has the potential to benefit from RL; however, further research attention should be directed towards parameter setting. In [40], the authors proposed an online RL approach for proactive process adaptations. Their work is placed in the context of proactive computing [41], aiming at eliminating the impact of upcoming issues during process execution based on prediction events.

Arguably, the most representative research work in the field of predictive process monitoring with the use of RL was proposed by [8]. The authors investigated the applicability of RL, and particularly the Deep Q Networks (DQN), for the prediction of both next event activity and time completion as well as the prediction of the whole progression of running cases. Their work represents the core of research on RL in predictive process monitoring. They compared their results to those derived from the application of LSTM and they demonstrated that DQN outperforms approaches based on LSTM architectures, while the plain workflow information seems to be insufficient to train an RL agent for the activity prediction task. The authors propose as a future research direction, among others, the use of alternative RL techniques.

## 3. The Proposed Approach for the Modelling and Predictive Monitoring of Business Processes

In this section, we present the proposed approach for the modelling and predictive monitoring of business processes under uncertainty with RL. The proposed approach takes as input an event log and provides as output predictions about the next activities, and specifically about activities that have been defined as goal states, along with the most efficient path on a process model. To do this, it incorporates five subsequent steps: (i) event log extraction (Section 3.1); (ii) process discovery for generating options in process models (Section 3.2); (iii) process statistical analysis for selection of the process model (Section 3.3); (iv) handling incomplete traces (Section 3.4); and (v) creating the uncertain process model and providing predictions about the business process (Section 3.5). Table 1 presents the data flow throughout the aforementioned steps by showing the inputs, the functions, and outputs of each step.

### 3.1. Event Log Extraction

**Figure 1:**
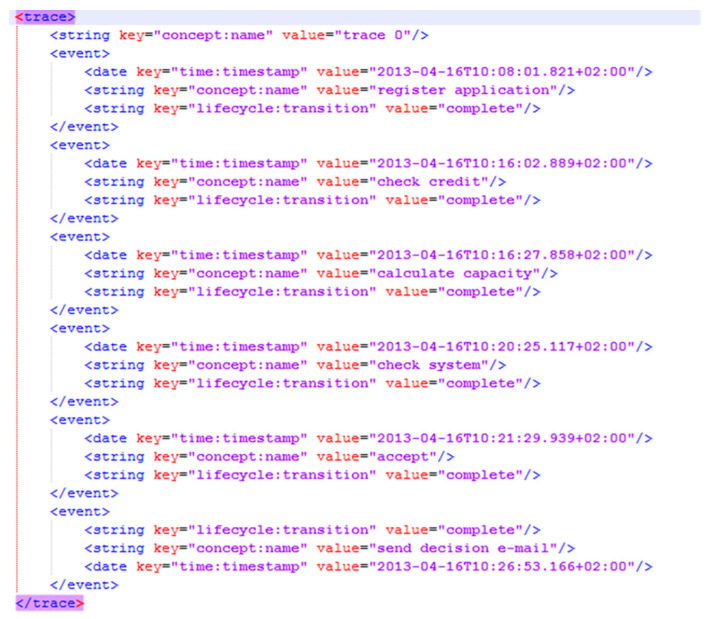
An example of the XES format.

To begin the proposed approach, the dataset is extracted from the relevant information system in the form of an event log, which is required to have certain attributes. These mandatory attributes include a case ID for each event, an activity related to each event, and timestamps to order events and measure performance. Optional attributes may also be included. However, event log data are not always in a usable format for process mining and may need to be reconstructed from other business data. Typically, event logs are in the XES format, which is a tag-based language for capturing system behaviors through event logs and streams. The XES standard includes a schema for the structure of the event log and its extensions, as well as prototypes for providing semantics to certain attributes [42]. Figure 1 provides an example of the XES format. It is also possible to convert the dataset into a CSV/XLS file for a better understanding of its elements.

### 3.2. Process Discovery for Generating Options in Process Models

Process discovery refers to a data-driven technique that generates a process model without prior knowledge by utilizing an event log as input. It must be able to cope with noisy, incorrect, and incomplete data. Process discovery is used in several ways such as understanding an unknown process structure, examining decision paths between choice points, identifying the path with the highest number of cases, and examining the distribution of cases along possible routes. Our methodology involves the implementation and comparison of three process discovery algorithms, namely Alpha Miner, Heuristic Miner, and Inductive Miner. For more details about process discovery algorithms, readers may refer to [43]. This step generates options for process models derived from the aforementioned process discovery algorithms. More specifically, it produces the following process models: Petri net derived from Alpha Miner, Petri net derived from Inductive Miner, Petri net derived from Heuristic Miner, Performance-based DFG, Frequency-based DFG, and Petri net derived from DFG. These process models feed into the subsequent step for further processing.

### 3.3. Process Statistical Analysis for Selection of the Process Model

The examination of a business process involves a statistical analysis that yields significant insights and serves as the basis for subsequent event log processing. Several metrics are employed to analyze and select the most suitable mining algorithm for the case, including [44] (i) fitness, which measures how well the model captures the event data; (ii) precision, which quantifies the fraction of the behavior allowed by the model that is not seen in the event log; (iii) generalization, which assesses the extent to which the resulting model will be able to reproduce future behavior of the process; and (iv) simplicity, which quantifies the complexity of the process model. The statistical measures help to determine the optimal process discovery algorithm and, thus, its resulting process model. Moreover, in this step, the frequencies of the activities and of the transitions among them are calculated, forming the basis for deriving the transition probabilities.

### 3.4. Handling Incomplete Traces

After exploring data, it is important to check if all the cases of the event log reach a final state. If not, it would prove problematic in the calculation of the transition probabilities among the activities of the process model. Therefore, we create a new state for all the cases that do not reach terminal states and terminate them in that state. We call this state “Frozen”. The transition probabilities are calculated using all individual instances on each state divided by the complete number of instances: $P\left(s\right)=\frac{instances(s)}{instances}$. Moreover, based on the frequencies of transitions among activities, calculated in the previous step, we calculate the transition probabilities that will be used for handling the uncertainty of the process model in the following step.

### 3.5. Creating the Uncertain Process Model and Providing Predictions about the Business Process

We use RL to solve an uncertain process model by finding the best policy. To do this, this step takes as input (i) the selected process model in order to utilize the activities as states and its structure with regard to the transitions among the activities; and (ii) the transition probabilities among the activities.

To apply RL, we require an environment and an agent. The environment consists of a set of states and actions, where the unique activities of the event act as states and the possible actions represent the selection of the next state. Rewards are specified by the developer based on what needs to be maximized. The agent is trained through the algorithm that interacts with the environment set. RL problems involve defining three sets: (i) the states of the environment (S), (ii) actions the agent can perform (A), and (iii) short-term rewardsI). During training, the agent learns to make sequential decisions, aiming to maximize the reward over an episode, which is a sequence of state, action, and rewards that concludes at a terminal state. The reward system is used during the training process to provide the agent with positive feedback for correct actions and negative feedback for wrong ones. Below are the key terms in RL models:

- Policy (π): defines the agent’s strategy to decide on the next action based on the present state.
- Discount factor γ (gamma): a number between 0 and 1 that determines the significance of future rewards. If γ is equal to or greater than 1 in a problem without a terminal state or when we cannot reach the terminal state, the undiscounted rewards may become infinite. If γ is 0, the agent only values short-term rewards, making it short-sighted.
- Value function (V): calculates the expected long-term reward with discount.
- Learning rate: determines the rate at which the agent overrides old knowledge with newly acquired knowledge.

Our proposed approach employs the Q-learning algorithm, a model-free reinforcement learning technique that learns the value of an action in a given state without requiring an environment model. It can handle problems with stochastic transitions and rewards without any adjustments [45]. Q-learning is based on temporal difference and approximates the optimal function q* using the action-value function *Q*, regardless of the policy being followed. To perform Q-learning, we need a Q-table, which is a matrix of state×action that stores the maximum expected future reward for the actions at each state. The Q-table can be initialized with a policy we are trying to improve or no policy.

During training, the agent can get stuck in certain actions, even if they are not the optimal ones, because of the exploration–exploitation problem. This problem can lead to sub-optimal policies and results. One approach to address this problem is the *ε*-greedy method, where *ε* is a parameter that controls the exploration–exploitation rate by determining the probability of choosing to explore or exploit. After the initialization, we select an action by using the *ε*-greedy method, where

$$0<\varepsilon <1\;\mathrm{and}\;p\;\mathrm{is}\;a\;\mathrm{random}\;\mathrm{probability}\;\mathrm{distribution}\;action=\left\{\begin{array}{c} random\;action\;(a)\;p<\varepsilon \\ maxQ(a)\;else\;1-\varepsilon \end{array}\right.$$

Then, we perform the chosen action and we evaluate the observed outcome and reward. Last, we update the Q-table. To carry out this procedure, the algorithm uses the Q-function (Sutton, and Barto, 2018).

$$Q\left(S_{t\;},A_{t}\right)\overset{}{\leftarrow }\underset{old\;value}{\underbrace{Q\left(S_{t\;},A_{t}\right)}}+\underset{learning\;rate}{\underbrace{a}}[\underset{new\;value\;with\;TD}{\underbrace{R_{t+1}+\gamma \underset{a}{\max}Q\left(S_{t+1},a\right)-Q\left(S_{t\;},A_{t}\right)]}}$$

To start the process, we set up the Q-table and let the agent select an action from a pool of available actions. Then, the agent collects rewards and updates the Q-table accordingly. To create the action pool, we use the reward matrix where non-negative rewards correspond to available actions and negative rewards correspond to unavailable actions. In order to randomly select an action from the pool, we use the Next Action function. Finally, we update the Q-table using the Q-function. This process is repeated over a number of episodes to train the agent.

The outcomes of this step, and thus the final outputs of the proposed approach, are (i) the most efficient path on the selected process model; and (ii) predictions about the next activity and the goal state, e.g., an activity that corresponds to a decision as the outcome of the process.

## 4. Implementation and Deployment in the Banking Sector

### 4.1. Technology Stack

The methodology described was implemented using Python programming language, a commonly used language for machine learning and data analytics projects. Anaconda was selected for its user-friendly interface, which includes the Spyder IDE and Jupyter notebook, as well as its ability to manage different environments. To read XES files, perform process discovery, and create process models in the form of Petri nets and DFGs, pm4py, a process mining library, was used. Data exploration, statistics, and permutations were performed using Pandas, which was also used in conjunction with numpy and pm4py. The probabilities for the Markov models were calculated using information extracted from the dataset. The matplotlib library was used to create visualizations such as plots and figures. NetworkX is a package designed for creating and analyzing complex networks and data structures like graphs. It was used to visualize the Markov models. TensorFlow is a collection of tools and libraries for machine learning development, and keras is an API for deep learning built on top of TensorFlow. Keras-rl, which integrates with keras, implements reinforcement learning algorithms and works with OpenAI Gym. These libraries were used to create the ANN and DQN agents. Gym, an open-source library developed for creating and evaluating reinforcement learning algorithms, was used to create a custom environment and manage experimentation and exploration. It is also compatible with libraries like TensorFlow.

### 4.2. Application of the Proposed Approach

#### 4.2.1. Event Log Extraction

The dataset used comes from the Business Process Intelligence (BPI) Challenge 2017 and specifically the Offer Event log. In this case, the dataset is already extracted from a database and it has been transformed to an event log, making it capable of subsequent processing. This event log pertains to a loan application process of a Dutch financial institute. This event log provides all the applications filled in 2016, and contains 1,202,267 events pertaining to 31,509 loan applications. For these applications, a total of 42,995 offers were created. We will be focusing on the business process that manages the 42,995 offers. The dataset’s original form was in XES but it was also converted into a csv/xls file in order to obtain a better understanding of the dataset and its elements. The features of the csv file are presented in Table 2. Table 3 presents the identified states that correspond to the activities of the business process along with their description.

#### 4.2.2. Process Discovery for Generating Options in Process Models

**Figure 2:**
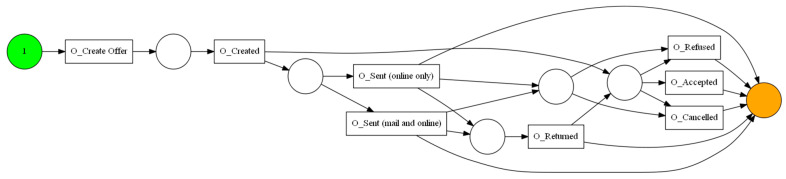
The Petri net derived from Alpha Miner.

**Figure 3:**
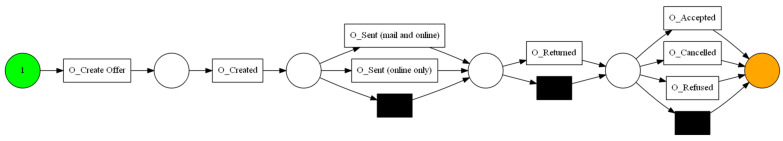
The Petri net derived from Inductive Miner.

**Figure 4:**
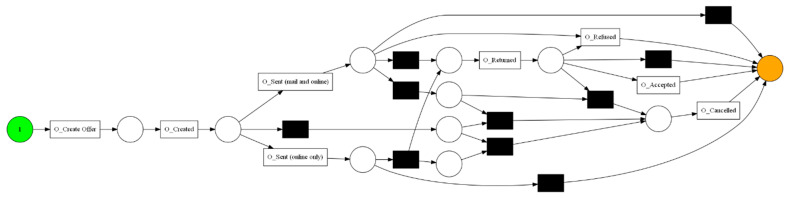
The Petri net derived from Heuristic Miner.

**Figure 5:**
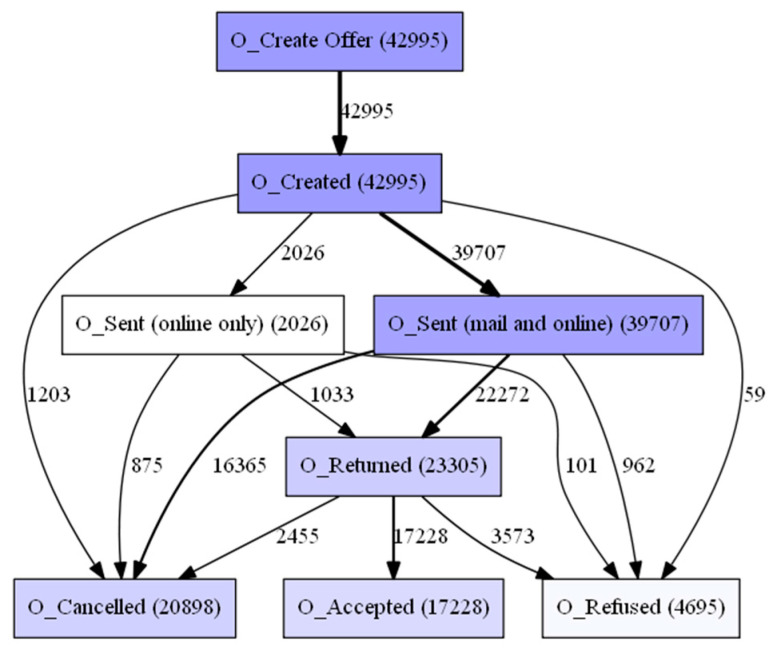
Performance-based DFG.

**Figure 6:**
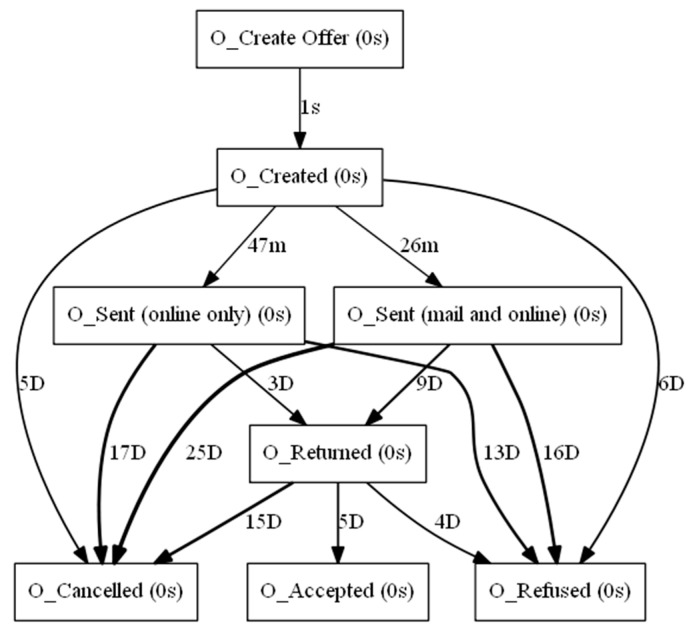
Frequency-based DFG.

**Figure 7:**
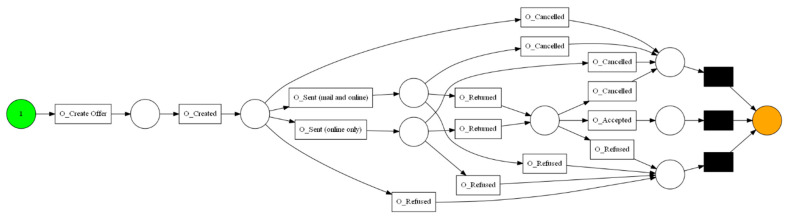
The Petri net derived from DFG.

The aforementioned event log feeds into the process discovery methods that were mentioned in Section 3.2. In this step, three algorithms and two types of visualization were used. The algorithms used are Alpha Miner (Figure 2), Inductive Miner (Figure 3), and Heuristic Miner (Figure 4). As for visualization, the following graphs (Figure 5 and Figure 6) and Petri nets (Figure 7) were used. A process tree can be directly transformed into a Petri net.

#### 4.2.3. Process Statistical Analysis for Selection of the Process Model

**Figure 8:**
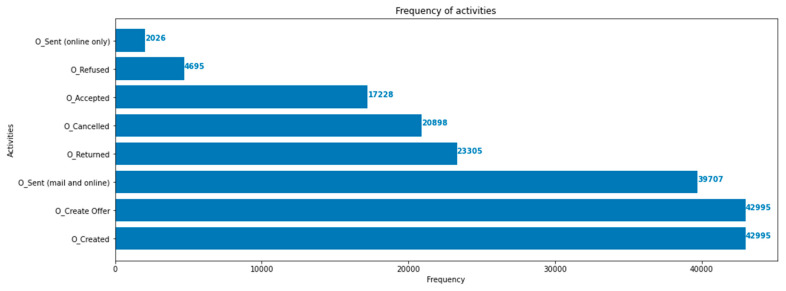
Bar chart with the frequency of activities in the event log.

The results obtained from calculating the percentage fit traces, average trace fitness, log fitness, precision, generalization, and simplicity are presented in Table 4. Based on these results, the Inductive Miner is selected as the preferred method for further processing due to its superior performance. The Inductive Miner produces process models that correspond to sound, block-structured workflow net systems, and is capable of handling infrequent behavior by allowing for multiple variants. Additionally, Figure 8 displays a bar chart indicating the frequency of activities, which will aid in calculating the transition probabilities.

#### 4.2.4. Handling Incomplete Traces

The aforementioned analysis revealed that some cases never reached a terminal state. Therefore, we created a new state called “Frozen” for all unfinished cases. We also noticed that all the cases from the “O_Created_Offer” state transitioned to the “O_Created” state, so we removed “O_Created_Offer” to eliminate the complexity of the process model. Moreover, we calculated the transition probabilities that will be used for handling the uncertainty of the process model in the following step.

#### 4.2.5. Creating the Uncertain Process Model and Providing Predictions about the Business Process

This step takes as input the Petri net derived from the Inductive Miner and the transition probabilities that were calculated in the previous step. In this way, it learns the environment of the RL model, i.e., its structure and the transition probabilities among its states. The reward matrix of the RL model is presented in Table 5, with the rows and columns representing the activities of the process model that was chosen. Each cell in the matrix corresponds to either a state or a transition between states. If an element has a value of minus one (“−1”), it indicates that there is no direct connection between the states. On the other hand, an element with a value of zero (“0”) denotes a direct connection between the states, making it a valid action to move to that state. The goal state is represented by the value one hundred (“100”), along with the transition to that state. The next step involves initializing the Q-matrix, which is of the same size as the reward matrix, i.e., 8 × 8.

The Q-matrix initialization is the next step, and it is of the same size as the reward matrix, 8 × 8. To implement Q-learning, three functions are used:

- Available_actions: This takes a number matching a state as input, which corresponds to an activity. The available actions for the input state are selected from that row, which are all indexes whose elements are non-negative.
- next_action: this takes the list of available actions as input and randomly selects one of them.
- learn: This has three inputs, including the current state, an action, and the discount factor gamma. The Q function is implemented, and a greedy method is used to select an action.

**Figure 9:**
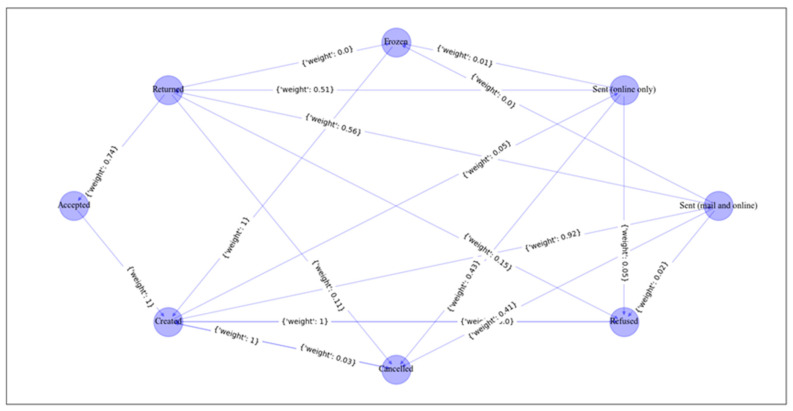
Visualization of the transitions among the states.

**Figure 10:**
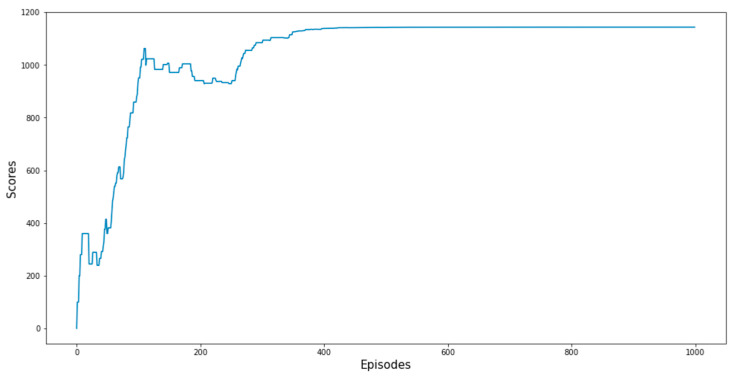
Q-learning training scores.

**Figure 11:**
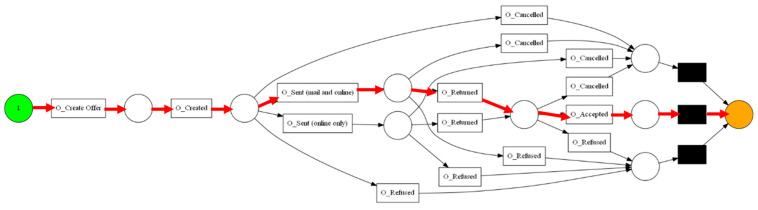
The most efficient path on the Petri-net.

The transitions among the states of the RL model are shown in Figure 9, which allows us to make predictions about the next activity during the runtime of a process instance. In this context, it also allows to perform predictions on the goal state which corresponds to a decision within the process, i.e., whether an application is accepted (O_Accepted) or rejected (O_Refused). The training graph in Figure 10 shows that it takes about 500 episodes of training to achieve the maximum score and stable results. Table 6 presents the results of Q-learning after training. Based on these results, the most efficient path on the process model is O_Created—O_Sent (mail and online)—O_Returned—O_Accepted, as shown in the Petri net of Figure 11.

### 4.3. Comparative Analysis with Deep Learning Extensions

In response to the increasing usage of deep learning techniques for predictive business process monitoring, we have replaced Q-learning with its deep learning counterpart in our proposed approach for comparison purposes. Deep Q-learning makes use of an Artificial Neural Network (ANN) to replace the Q-table, creating a Deep Q-Network (DQN). Instead of value iteration, this approach employs a function approximator to obtain an estimate of the optimal Q-function. To overcome this challenge, we need to introduce the concepts of experience replay and target network. The agent stores its experiences in memory through experience replay. Once a certain memory threshold is reached, the agent can learn from it by randomly selecting uniformly distributed samples from the stored memory, learning from batches to prevent biased decisions. The target network, on the other hand, adds stability to the training process. The second network generates the target Q values used to calculate the loss for each action during training. The updating of the target network should be frequent yet slow.

The use of deep Q-learning algorithms for predictive business process monitoring has been shown to require a large amount of data for optimal performance, according to previous research [46,47]. In our proposed approach, we first create a network and a target network, and then choose actions based on the exploration–exploitation trade-off. The network’s weights are updated after each action selection [48]. Unlike traditional DQN, which uses three convolution layers with ReLU activation for image inputs, we use four dense layers with ReLU and linear activation for numerical input data. To set up the reinforcement learning environment and tasks, we use the Gym library and create an environment represented as a class. We then set up a reward matrix or step function based on our goal, guiding the agent through the matrix to learn optimal paths. After all necessary attributes are set up, we validate the agent’s actions, set rewards for state transitions, and notify the agent of cycle completion or incomplete transitions.

**Figure 12:**
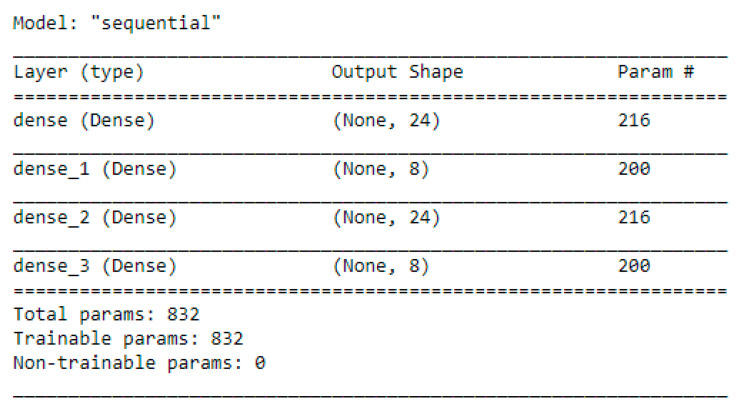
ANN model description.

To calculate and select rewards for each action, we utilize a reward matrix based on the same principles as Q-learning (see Table 7.). The architecture of the Artificial Neural Network we employ is shown in Figure 12. Our agent relies on the ANN along with an *ε*-greedy policy and sequential memory.

After undergoing 50,000 training iterations, the agent has identified the optimal route, which comprises three steps and achieves a maximum reward of 102. Furthermore, we integrated a binary sorting neuron that utilizes a refined dataset and accepts seven inputs, five of which represent the primary data for each case, while the remaining two signify the classification outcomes. These primary elements include:

- Main elements:
  ○ case: MonthlyCost
  ○ case: FirstWithdrawalAmount
  ○ case: CreditScore
  ○ case: OfferedAmount
  ○ case: NumberOfTerms
- Classification Results:
  ○ Selected
  ○ Accepted

To begin, we trained the ANN with both the primary elements and classification outcomes. Following the completion of training, we leveraged the ANN to categorize each case based on the five primary elements and then contrasted its own forecasts with the actual classifications (as illustrated in Table 8).

## 5. Conclusions and Future Work

Process mining is a useful method for analyzing business processes by examining their observed behavior in event logs. However, traditional process mining methods have their limitations due to their complexity and lack of ability to handle uncertainty and make predictions. Recent research has focused on developing predictive approaches for business process monitoring, and this paper proposes a new approach using reinforcement learning (RL). The proposed RL-based approach was evaluated in a use case from the banking sector and compared to traditional Q-learning and DQN methods. The results show that Q-learning has better performance in simple problems, while DQN is more suitable for complex problems due to its neural network structure.

Our future work will move in the following directions. First, we will embed the hereby proposed approach for predictive business process monitoring into the “Smyrida” system that we have developed [12]. “Smyrida” is a modular software system in the form of a web application with open APIs, making it adaptive to new techniques for process mining. Second, we plan to incorporate and compare additional RL algorithms in order to examine their benefits, and also compare the proposed approach with other ML and deep learning algorithms.

## Figures and Tables

**Table 1:** The inputs, functions, and outputs of each step of the proposed approach.

Section	Step	Input	Function	Output
***3.1***	***Event Log Extraction***	Information System Database	Transformation into event log in CSV or XES format	Event log in CSV or XES format
***3.2***	***Process Discovery for Generating Options in Process Models***	Event log in CSV or XES format	Process discovery Alpha Miner Heuristic Miner Inductive Miner	Petri net from Alpha Miner Petri net from Inductive Miner Petri net from Heuristic Miner Performance-based Directly Follows graph (DFG) Frequency-based DFG Petri net from DFG
***3.3***	***Process Statistical Analysis for Selection of the Process Model***	Petri net from Alpha Miner Petri net from Inductive Miner Petri net from Heuristic Miner Performance-based DFG Frequency-based DFG Petri net from DFG	Calculation of evaluation metrics: Fitness Precision Generalization Simplicity Selection of the optimal process discovery algorithm and process model Calculation of the frequencies of activities and transitions	Selected process model Transition probabilities among activities
***3.4***	***Handling Incomplete Traces***	Selected process model Event log	Identification of incomplete traces Creation of a “Frozen” state for incomplete traces Calculation of transition probabilities among activities based on frequencies	Transition probabilities among activities
***3.5***	***Creating the Uncertain Process Model and Providing Predictions about the Business Process***	Selected process model Transition probabilities among activities	Creation of the uncertain process model using RL Definition of the goal state Calculation of the optimal policy	The most efficient path on the selected process model Predictions about the next activity and the goal state

**Table 2:** The elements of the dataset.

Element Name	Description
Action	Action taken in the business process.
Org:resource	User/actor from the organization.
Concept:name	Business process state name.
EventOrigin	Origin of business process (offer).
EventID	The unique identifier of the event.
Lifecycle:transition	Transition of state (complete).
Time:timestamp	Given time at each state.
Case:concept:name	The unique identifier of the event.
Case:MonthlyCost	The monthly costs to be paid by the customer to reimburse the loan.
Case:Selected	Boolean that indicates whether an offer is signed by the customer or not.
Case:ApplicationID	The identifier of the application.
Case:FirstWithdrawalAmount	The initial withdrawal amount.
Case:CreditScore	The credit score of the customer. The higher the credit score, the higher the client trustworthiness.
Case:OfferedAmount	The loan amount offered by the bank.
Case:NumberOfTerms	The number of payback terms.
Case:Accepted	The offer is acceptable based on the bank’s terms.
OfferID	The unique identifier of the offer.

**Table 3:** The activities of the event log.

State Name	Description
O_Create offer	Creating a credit offer.
O_Created	Offer created.
O_Sent (online only)	Offer sent online.
O_Sent (mail and online)	Offer sent online and by mail.
O_Returned	Client submitted documents for the offer.
O_Accepted	Application passed all checks and verification.
O_Cancelled	Offer canceled by the client.
O_Refused	Offer canceled by the bank.

**Table 4:** Process statistical analysis results of the process discovery algorithms.

	Alpha Miner	Inductive Miner	Heuristic Miner
**Percentage fit traces**	0.0	100.0	38.311
**Average trace fitness**	0.839	1.0	0.909
**Log fitness**	0.835	1.0	0.914
**Precision**	0.812	0.780	1.0
**Generalization**	0.991	0.983	0.799
**Simplicity**	0.455	0.630	0.577

**Table 5:** Q-learning reward matrix.

	Frozen	O_Accepted	O_Canceled	O_Created	O_Refused	O_Returned	O_Sent (Mail and Online)	O_Sent (Online Only)
Frozen	−1	−1	−1	0	−1	−1	−1	−1
O_Accepted	−1	100	−1	0	−1	−1	−1	−1
O_Canceled	−1	−1	−1	0	−1	−1	−1	−1
O_Created	−1	−1	0	−1	0	−1	0	0
O_Refused	−1	−1	−1	0	−1	−1	−1	−1
O_Returned	0	100	0	−1	0	−1	−1	−1
O_Sent (mail and online)	0	−1	0	−1	0	0	−1	−1
O_Sent (online only)	0	−1	0	−1	0	0	−1	−1

**Table 6:** Q-learning results.

	Frozen	O_Accepted	O_Canceled	O_Created	O_Refused	O_Returned	O_Sent (Mail and Online)	O_Sent (Online Only)
Frozen	0	0	0	51.2	0	0	0	0
O_Accepted	0	100	0	51.2	0	0	0	0
O_Canceled	0	0	0	51.2	0	0	0	0
O_Created	0	0	40.96	0	40.96	0	63.99	63.99
O_Refused	0	0	0	51.2	0	0	0	0
O_Returned	40.96	100	40.96	0	40.96	0	0	0
O_Sent (mail and online)	40.96	0	40.96	0	40.96	79.99	0	0
O_Sent (online only)	40.96	0	40.96	0	40.96	80	0	0

**Table 7:** DQN reward matrix.

	Frozen	O_Accepted	O_Canceled	O_Created	O_Refused	O_Returned	O_Sent (Mail and Online)	O_Sent (Online Only)
Frozen	−1	−1	−1	0.1	−1	−100	−1	−1
O_Accepted	−1	−1	−1	0.1	−1	−100	−1	−1
O_Canceled	−1	−1	−1	0.1	−1	−100	−1	−1
O_Created	−1	−1	−1	0.1	−1	−100	1	1
O_Refused	−1	−1	−1	0.1	−1	−100	−1	−1
O_Returned	−1	100	−1	−1	−1	−100	−1	−1
O_Sent (mail and online)	−1	−1	−1	−1	−1	1	−1	−1
O_Sent (online only)	−1	−1	−1	−1	−1	1	−1	−1

**Table 8:** DQN accuracy.

	Accuracy
First training results (case: Selected)	84.85%
Second training results (case: Accepted)	67.57%

## Data Availability

The dataset that was used was retrieved from the BPI Challenge 2017 (https://data.4tu.nl/articles/dataset/BPI_Challenge_2017/12696884, accessed on 31 July 2023).
